# Whether or Not the Effects of *Curcuma longa* Supplementation Are Associated with Physical Exercises in T1DM and T2DM: A Systematic Review

**DOI:** 10.3390/nu13010124

**Published:** 2020-12-31

**Authors:** Ailton Santos Sena-Júnior, Felipe José Aidar, Ana Mara de Oliveira e Silva, Charles dos Santos Estevam, Carla Roberta de Oliveira Carvalho, Fábio Bessa Lima, Jymmys Lopes dos Santos, Anderson Carlos Marçal

**Affiliations:** 1 Graduate Program in Physical Education, Universidade Federal de Sergipe, São Cristóvão, Sergipe CEP 49100-000, Brazil; juniorsena_nutri@hotmail.com (A.S.S.-J.); fjaidar@gmail.com (F.J.A.); jymmyslopes@yahoo.com.br (J.L.d.S.); 2 Group of Studies and Research of Performance, Sport, Health and Paralympic Sports—GEPEPS, Federal University of Sergipe, São Cristóvão, Sergipe CEP 49100-000, Brazil; 3 Graduate Program in Physiological Sciences, Federal University of Sergipe, São Cristóvão, Sergipe CEP 49100-000, Brazil; cse.ufs@gmail.com; 4 Nutrition Sciences Graduate Program, Federal University of Sergipe, São Cristóvão, Sergipe CEP 49100-000, Brazil; anamaraufs@gmail.com; 5 Department of Nutrition, Federal University of Sergipe, São Cristóvão, Sergipe CEP 49100-000, Brazil; 6 Health Sciences Graduate Program, Federal University of Sergipe, Aracaju, Sergipe CEP 49060-100, Brazil; 7 Postgraduate in Biotechnology, Northeast Network in Biotechnology (RENORBIO), Federal University of Sergipe, São Cristóvão, Sergipe CEP 49100-000, Brazil; 8 Department of Physiology, Federal University of Sergipe, São Cristóvão, Sergipe CEP 49100-000, Brazil; 9 Department of Physiology and Biophysics, Institute of Biomedical Sciences (ICB), University of São Paulo, São Paulo CEP 05508-000, Brazil; croc@fisio.icb.usp.br (C.R.d.O.C.); assebfabio@gmail.com (F.B.L.); 10 Department of Morphology, Federal University of Sergipe, São Cristóvão, Sergipe CEP 49100-000, Brazil

**Keywords:** diabetes, saffron, turmeric, supplementation, physical exercise

## Abstract

Diabetes mellitus is one of the most prevalent chronic diseases in the world; one of its main characteristics is chronic hyperglycemia. Pharmacotherapy and other alternatives such as regular exercise are among the therapeutic methods used to control this pathology and participate in glycemic control, as well as the ingestion of plant extracts with antioxidant effects. Among the different plants used for this purpose, curcumin has potential to be used to attenuate the hyperglycemic condition triggered by diabetes mellitus (DM). Some prior studies suggest that this plant has antioxidant and hypoglycemic potential. This review aims to evaluate the antioxidant and hypoglycemic potential of curcumin supplementation in Type 1 DM (T1DM) and Type 2 DM (T2DM). The search considered articles published between 2010 and 2019 in English and Portuguese, and a theoretical survey of relevant information was conducted in the main databases of scientific publications, including the Virtual Health Library and its indexed databases, PubMed, LILACS (Latin American and Caribbean Literature on Health Sciences—Health Information for Latin America and the Caribbean—BIREME/PAHO/WHO), and Scientific Electronic Library Online (SciELO). The associated use of turmeric and physical exercise has demonstrated antioxidant, anti-inflammatory, and hypoglycemic effects, suggesting that these could be used as potential therapeutic methods to improve the quality of life and survival of diabetic patients.

## 1. Introduction

Diabetes mellitus (DM) is a metabolic disorder of multiple etiologies; the main factors involved in the development of this pathogenesis are genetic disorders, environmental risk factors, and/or autoimmune disease [1]. DM is mainly characterized by chronic hyperglycemia, in which there is marked elevation of blood glucose due to the absence or ineffectiveness of insulin hormone action on cell receptors [2,3,4].

This disease is one of the most prevalent chronic diseases in the world and one of the greatest public health challenges of the 21st century, and its incidence is increasing in both underdeveloped and developing countries [5,6,7]. It is estimated that the number of diabetic individuals aged 20–79 years was 8.8% in 2015, corresponding to 415 million people. If this trend continues, the number of people with diabetes in the year 2040 is expected to reach 642 million [8,9].

Type 1 diabetes mellitus (T1DM) is less prevalent than Type 2 diabetes mellitus (T2DM) and is considered an inflammatory and autoimmune pathology due to the impairment of insulin production caused by the destruction of pancreatic β cells resulting from the infiltration of autoreactive T lymphocytes in the endocrine pancreas [10]. T2DM is becoming increasingly prevalent, accounting for about 90% of cases in the world’s population, and is characterized by resistance to the action of insulin associated to hyperinsulinemia during the postprandial period. Thus, hyperglycemia is the most evident symptom in this pathology [8,11].

Among the several therapeutic methods used to treat and control variations in blood glucose levels in humans and in experimental models with T1DM and T2DM, the most common are as follows: medication (with the use of hypoglycemic agents associated or not associated with the administration of exogenous insulin), adequate and healthy eating, regular physical activity, and/or physical exercise [12,13,14]. The adequate control of glucose levels near to reference values is necessary for health maintenance, reducing the possible development of other diseases associated to both types of diabetes such as retinopathy, cardiopathy, nerve and kidney damage, as well as other diseases caused by oxidative stress in uncontrolled diabetics’ conditions [15].

Physical activity is essential for glycemic control and the reestablishment of the body’s antioxidant defense in patients with diabetes [12]. Physical exercise has been associated with a protective factor for health since the 1950s. Since then, its benefits have been associated with a reduction in chronic diseases, weight reduction in adults, and reduced risk of premature death from cardiovascular diseases [16,17,18].

In addition to the pharmacological prescription for drug use, dietary supplementation with antioxidant compounds has also shown promising results on the maintenance of blood glucose in altered physiological conditions [19,20,21]. Among these dietary supplements, turmeric (*Curcuma longa*), which contains natural phenolic compounds widely used in foods, beverages, and medications, appears to act beneficially on glycemic control, attenuating hyperinsulinemia and homeostasis model assessment index (HOMA-IR) and delaying the onset of the comorbidities often found in patients with diabetes [22,23,24].

Some researchers have evaluated the effects of exercise after antioxidant-rich supplementation to determine the possible protective effect of the intake of natural supplements on muscular damage and oxidative stress in skeletal muscle after exercise training [19,25]. These effects are partly due to exercise type, frequency, and intensity [26].

The present study aimed to evaluate, through a literature review, the effects of turmeric consumption in the form of an extract and the practice of various types of physical exercise found in the literature on glycemic control and chronic complications in humans and experimental models with T1DM and T2DM.

## 2. Methodology

The methodological approach of this review followed article search strategies and inclusion criteria, including data collection and an analysis phase. This systematic review followed the guidelines of the Preferred Reporting Items for Systematic Reviews and Meta-Analysis (PRISMA) [27]. The protocol was registered with Centre of Reviews Dissemination (CRD) number 154,729 in the International Prospective Register of Systematic Reviews (PROSPERO).

### 2.1. Search Strategy and Inclusion Criteria

This systematic review considered articles published between 2010 and 2019 in English and Portuguese. The PICO (Patient, Intervention, Comparison and Outcomes) strategy was used, considering studies with rodent and human diabetics that performed physical activity with the use of long *Curcuma longa*/curcumin supplementation (P = patient), evaluated on performance of the activity. Supplementation (I = intervention) is meant to attenuate the pathology of diabetes (C = comparison of intervention or control), with the objective of verifying physical exercise capacity together with the supplementation of *Curcuma longa*/curcumin in the control of diabetes mellitus (O = outcome) using the following guiding question: “What are the effects of *Curcuma longa* supplementation associated with exercise in patients and experimental models with diabetes?”.

**Figure 1:**
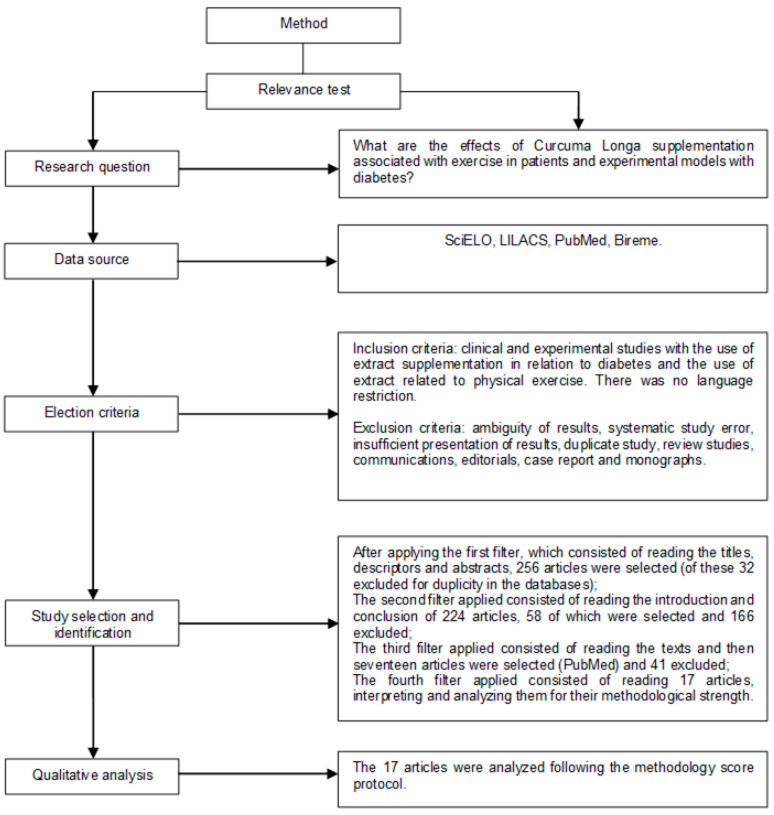
Research strategy flowchart and article selection.

The study design and the eligibility criteria are shown in Figure 1. For studies to be considered preliminarily eligible, full texts were evaluated to verify that they met all the inclusion criteria. The inclusion criteria were scientific studies on the subject that showed evidence of turmeric’s effect on animal and human models.

The exclusion criteria were ambiguous results, duplicates of database-based studies, review studies, communications, case reports, summaries of scientific meetings, monographs, comments, or editorials.

Keywords were selected from the Health Sciences Descriptors of the Virtual Health library (VHL) and Medical Subject Headings (MeSH) (PubMed) to identify relevant studies in the PubMed, SciELO, and LILACS. Descriptors were “physical activity”, “physical exercise”, “turmeric”, and “diabetes”. This research was conducted from December 2017 to February 2019.

### 2.2. Evaluation Validity and Data Extraction Process

After obtaining the list of studies with the chosen descriptors, the relevance test was applied and each study was carefully analyzed by two eligibility reviewers who independently conducted the research and decided in a consensual manner which studies would be selected. In the case of divergence of results, a third reviewer was consulted to resolve the divergence, as suggested in the literature.

Initially, article titles, descriptors, and abstracts were identified; the first search filter was applied to select them. Subsequently, based on the results obtained, the second filter was applied by reading the introduction and conclusion sections. If the article was considered eligible, the article was read in full and the third filter was applied, which consisted of reading the articles by peers, resulting in seventeen articles being selected for the next stage of analysis.

In the fourth filter, the articles were analyzed and interpreted according to their methodological strength. The review was performed blindly to authors and journals to avoid any selection bias and possible conflicts of interest. The seventeen remaining articles were included in our work.

Studies were summarized and presented in a table that is reported in the results section (according to flowchart) containing the following information: author and year of publication, model, supplementation dosage and duration and results. Data were qualitatively analyzed, since the methodology used in the research was heterogeneous. As for the methodological strength, 100% of the final articles had moderate strength (considering the journal’s impact, clinical and experimental studies). The *p*-value adopted in all articles was *p* < 0.05.

## 3. Results and Discussion

### 3.1. Turmeric Glycemic Control and Insulin Sensitivity

Some authors have been increasingly committed to identifying medicinal plants, their effects for the treatment of diseases, and their possible application in primary health care [28,29,30].

In this context, turmeric (*Curcuma longa* L.) is present on the National List of Medicinal Plants of Interest to Brazilian Unified Health System, being widely used in the world cuisine as a natural compound as well as in therapeutic activities through popular knowledge recognized by the National Health Service of Brazil [31]. Curcuminoid, extracted from *Curcuma longa* rhizomes, is the main active compound of turmeric, and its yellow color is indicative of its biological effects [32,33].

Treatment with curcumin, either in its solubilized form in ethanolic extracts, incorporated in carboxymethylcellulose or in water is promising against diabetes due to its effects on glycemic control. Among them, the use of curcumin promotes a reduction in the glycated hemoglobin concentration and, consequently, a reduction in the plasma glucose concentration, indicating its potential as a drug for glycemic control [34].

There are several reports in the literature indicating a wide variety of pharmacological activities for *Curcuma longa*, proving anti-inflammatory, antiviral, antibacterial, antioxidant, antifungal, and anticarcinogenic effects, among other therapeutic actions [23]. In the present review, among the articles selected, as shown in Table 1 and Table 2 (and other studies, as follows below), the use of the oral supplementation of turmeric at a dose of 100 mg/kg and weight of 200 mg/kg for sixteen weeks promoted a reduction in the concentration of blood glucose and diabetes-induced attenuation of body-weight loss, as well as a strong antioxidant capacity in the retina of diabetic rats. An antiapoptotic effect was observed by increasing the expression of *B-cell lymphoma protein 2* (Bcl-2) and the downregulation of *associated protein X* (Bax) expression in the retina of diabetic rats, concluding that curcumin has great potential in the treatment of diabetic retinopathy, which is probably attributed to its hypoglycemic and antioxidant effects [35].

Oral curcumin supplementation was effective at a dose of 100 mg/kg body weight for 8 weeks for improving hyperglycemia and restoring body weight in animal models with T1DM when compared to the diabetic group without supplementation. The spleen, considered as a peripheral immune organ, showed white pulp reductions and red pulp activation in the diabetic group; thus, curcumin treatment after diabetes induction restored and improved splenic tissue under conditions close to those of the control group [36].

Among the ways to evaluate circulating blood glucose, the oral glucose tolerance test (OGTT) is considered by the WHO as the ideal method for diagnosing T1DM and T2DM, both individually and in epidemiological studies. When glucose intolerance is diagnosed, it represents the initial pathophysiological condition of the T2DM pathogenesis and may contribute to the development of cardiovascular diseases [4].

Su et al. [37] assessed blood glucose with the OGTT and found that, after the fourth week of treatment, the blood glucose levels decreased 30 min after curcumin supplementation; until week 4, the blood glucose levels reduced between 60 and 120 min after supplementation compared to the other groups. At week 8, glycemic controls were observed in both groups. The insulin tolerance test showed that, in the same period, the fibrinogen-like (FBG) concentration in the curcumin group was lower than in the control group. Following subcutaneous insulin application, the blood glucose levels decreased between 40 and 90 min in the curcumin group. Research has shown that curcumin treatment after 8 weeks significantly improved metabolic parameters, such as increased insulin sensitivity and increased glucose tolerance. The same study showed that curcuminoid supplementation can reduce the serum atherogenic lipid levels in low-intensity lipoprotein cholesterol (LDL-C), total cholesterol (TC), and triglycerides (TG).

Other authors have also shown benefits related to a decrease in serum lipid levels, in which the combined therapy of curcuminoids (1000 mg/day) associated with piperine (10 mg/day) used for 12 weeks showed significant reductions in TC, LDL-C, TG, and lipoprotein C. An increase in the serum high-density lipoprotein HDL-C concentration was also found [38].

The American Diabetes Association recommends the use of glycated hemoglobin (HbA1c) as a method for diagnosing prediabetes, T1DM, and T2DM. HbA1c is the standardized dosing method defined by the International Expert Committee, considering type 1 and 2 diabetes values equal to or above 6.5%. HbA1c has many advantages over fasting blood glucose for diagnosis, especially at higher preanalytical stability and lower daily variation during periods of stress or disease [12].

Combination therapy (500 mg/day of curcuminoids coadministered with piperine 5 mg/day orally using capsules) for 3 months resulted in a significant reduction in serum glucose concentration [38]. In another study, C-peptide and HbA1c after curcuminoid supplementation were reduced compared with the placebo group [39]. These authors revealed beneficial effects regarding curcumin and piperine supplementation in glycemic parameters.

Diabetic rats which were orally supplemented with curcumin showed a reduction in plasma glucose concentration, plasma malondialdehyde concentration, and plasma glutathione peroxidase (GSH-Px), and catalase activity (CAT) was observed. However, the content of superoxide dismutase (SOD) and insulin increased [40]. In addition, the oxidative stress in diabetic rats can be attenuated by curcumin via the activation of the Keap1-Nrf2-ARE signaling pathway, as evidenced by the decrease in blood glucose concentration and increase in the transcription of antioxidant genes [38].

Most studies analyzing the relationship between *Curcuma longa* and T1DM/T2DM have shown that turmeric intake acts in the glycemic control and normalization of insulin resistance, effects which are partly due to molecular adjustments. Therefore, turmeric has potential as a form of adjuvant therapy for patients with T2DM and T2DM.

### 3.2. Intracellular and Antioxidant Effects of Curcumin

Compounds such as uric acid, ascorbic acid, reduced glutathione, α-tocopherol, sulfhydryl-containing molecules, CAT, SOD, and glutathione peroxicity participate in the body’s antioxidant defense systems [21,50]. The analysis of these molecules by biochemical tests has been recommended to elucidate the functional and structural abnormalities caused by diabetes, which are related to impaired endogenous antioxidant capacity. An increase in reactive species and free radicals, especially nitrogen and oxygen, is observed. The polyol pathway, involved in nonenzymatic glycation products, is related to hyperglycemia and has been used as a possible related marker to increase the free radical plasma concentration in patients with diabetes [51].

Curcumin supplementation may increase the expression of CAT, GSH-Px, heme oxygenase 1, and NADPH dehydrogenase 1 enzymes and decrease SOD1 expression, thus reducing oxidative stress [40]. In another study [41], twenty-four rats were divided into six groups—1 as control rats; 2 as diabetic rats; 3 and 4 as diabetic rats who received curcumin therapy of 200 and 400 mg/kg for 3 days, respectively; and 5 and 6 as diabetic rats who received curcumin treatment of 200 to 400 mg/kg for 8 days, respectively—to determine the SOD expression. A decrease in the SOD expression was observed in the diabetic group (without curcumin treatment). Curcumin treatment at doses of 200 and 400 mg/kg for 3 and 8 days led to significant differences in SOD expression compared to the diabetic group (without curcumin treatment). No significant differences were found between dose and the duration of SOD expression [41]. These same authors suggested that curcumin is an important antioxidant against oxidative stress in diabetes through SOD expression in cochlear fibroblasts.

The diabetic and obese rats were treated with a daily dose of curcumin, finding that curcumin treatment significantly reduced apoptosis in the testicular cells of rats [16]. Other authors also showed through molecular analysis that curcumin treatment significantly and simultaneously decreased oncogenic proteins that inhibit apoptosis to Bax and increased Bcl-2 expression, increasing the Bcl-2:Bax ratio. Curcumin treatment also significantly decreased the malondialdehyde (MDA) and increased the SOD concentration [35]. In conclusion, the ability of curcumin to inhibit oxidative stress and modulate the Bax/Bcl-2-mediated cell death pathway reveals its potential as a therapeutic agent against diabetes.

Curcumin treatment in the diabetic group protected cells from inflammatory and endoplasmic reticulum (ER) damage, as well as mitochondrial apoptotic death, suggesting that curcumin has the potential to act as a therapeutic antidiabetic, antioxidant, anti-inflammatory, and antiapoptotic agent against mediated splenic damage due to diabetes [36]. The findings indicate that orally supplemented curcumin promotes an improvement in collagen deposition in the cardiac tissue of diabetic rats [42]. These same authors showed an increase in the deposition of type I and type III collagen in cardiac tissues, accompanied by a marked reduction in the production of transforming growth factor β1 (TGF-β1). These results demonstrate the beneficial effect of curcumin on collagen synthesis in diabetic rats, as reported by other authors [43,44].

In this sense, these studies demonstrate that curcumin can attenuate oxidative stress, the effects of which are partly due to the increased expression and/or activity of antioxidant enzymes that can attenuate mitochondrial dysfunction and liver damage and reduce inflammatory processes.

### 3.3. Physical Exercise and Curcuma

Physical exercise is characterized by the repetition of directed movements, with the increase in oxygen consumption caused by the recruitment of muscle fibers at the moment of the movement action [52]. Exercise is a subgroup of physical activity in an elaborate and oriented manner to maintain physical fitness, which can be defined as any muscle movement that results in strength [53].

The regular practice of physical activity when designed as an adequate training program, respecting each practitioner’s biological individuality with appropriate intensity, duration, frequency, and progression, will result in benefits to components related to the organism’s functional health. Thus, it can prevent and/or mitigate the effects of degenerative chronic diseases such as hypertension, diabetes, obesity, arthrosis, osteoporosis, dyslipidemia, and metabolic syndrome, among others [54].

There are few studies evaluating the effects of physical exercise associated with turmeric supplementation. The effects of oral curcumin ingested before and after eccentric exercise on muscle injury and inflammation markers in healthy men who ingested 180 mg/day of curcuma for 7 days were observed with a decrease in IL-8 after 12 h of physical exercise [45]. Creatine kinase (CK) activity was also lower between 3 and 6 days and 5 and 7 days after exercise. Thus, curcumin ingestion after exercise can attenuate muscle damage and facilitate faster recovery.

In another study, it was found that curcumin intake and aerobic exercise training increase flow-mediated dilation in women, both improving age-related decline and endothelial function. The women, who ingested 150 mg/day of turmeric and underwent aerobic exercise training for 8 weeks, showed an increase in flow-mediated endothelial dilation, while no changes were observed in the control group [46].

By relating physical exercise to dietary planning, healthy male athletes who received a Mediterranean diet and supplementation with curcumin and *Boswellia serrata* (BSE) after 12 weeks of exercise showed decreased nonesterified fatty (NEFA), MDA, and total soluble form of receptor for advanced glycation end-products (sRAGE) in the supplemented group [47]. Therefore, supplementation with curcumin and BSE demonstrates positive effects on chronic glycosylation and lipid peroxidation in athletes.

For the men who received oral curcumin supplementation at a dose of 2.5 g twice daily two days before and three days after eccentric exercise, it was identified that between 24 and 48 h after exercise, curcumin was able to reduce moderate exercise pain and small reductions in creatine kinase activity [26]. In the same study, they found that curcumin increased the interleukin-6 concentrations at 0 and 48 h from baseline. However, the supplementation decreased IL-6 at 24 h after exercise. Therefore, curcumin consumption probably promotes greater efficiency in recovery and muscle performance after training.

In the study by Sugawara et al. [48], female subjects were divided into four interventions: placebo intake, curcumin intake, placebo intake plus exercise, and curcumin intake plus physical exercise. A curcumin or placebo dose (150 mg/day) was administered for 8 weeks. They observed that after interventions, systolic blood pressure (SBP) significantly decreased in both groups, whereas aortic SBP significantly decreased only in treatment with combined exercise and curcumin supplementation. These studies suggest that regular endurance exercise associated with daily curcumin intake may reduce left ventricular afterload to an extent greater than monotherapy with any single intervention.

Takahashi et al. [49] conducted a study in which male participants were divided into three groups: control (placebo), isolated (only before exercise), and double (before and immediately after exercises with curcumin supplementation). Each subject received the oral administration of 90 mg curcumin or placebo 2 h before exercise and immediately after exercise. Reactive oxygen metabolites such as reactive oxygen metabolites (ROMs) and TRX-1 measured after exercise were significantly higher than pre-exercise values. Serum biological antioxidant potential assessed by plasma thiobarbituric acid reactive substances (TBARS) and concentrations measured immediately after exercise were significantly elevated in the curcumin supplementation group compared with the pre-exercise values. These results suggest that curcumin supplementation may attenuate the stress-induced oxidation caused by exercise, increasing the antioxidant effect.

Exercise can promote beneficial adjustments in aerobic capacity and lipid and glycemic control, as it controls insulin and glucose homeostasis, promotes increased fatty acid oxidation in muscles, and reduces blood glucose concentration, in addition to attenuating systemic inflammation and improving immune cell function [55,56].

Given the above, physical exercise can be used as an important therapeutic treatment for diabetes because it is able to increase glucose uptake by skeletal muscle, using the insulin-independent pathway. In addition, physical exercise associated with *Curcuma longa* supplementation improves mitochondrial activity and antioxidant defenses, thus reducing oxidative stress.

### 3.4. Toxicity, Adverse Effects, and Contraindication

Studies evaluating oral toxicity in rats by the continuous use of up to 0.5 g/kg *Curcuma longa* L. essential oil for 13 weeks and curcumin up to 10,000 ppm for 70 days did not show signs of toxicity, death, and/or organ changes [57]. In humans, turmeric consumption toxicity has no shown adverse effects, even at the maximum dose of 8000 mg/day for up to 3 months [58,59]. In another study, the excessive consumption of diferuloylmethane (a component of curcumin) may cause nausea and gastrointestinal irritation in a few individuals of the experimental group [59,60].

According to the Food and Agriculture Organization/World Health Organization committee, among which one of their activities is the assessment of toxicity for food additives and seasonings, the acceptable daily intakes of curcumin is 0 to 3.0 mg/kg body weight according to the values of the no-observed-effect level of 250 to 320 mg/kg body weight per day obtained in experimental models [58,59]. Additional long-term studies are needed to further elucidate associations between turmeric intake/supplementation and risk of toxicity.

In addition, results found in studies have shown that the use of *Curcuma longa* can mitigate various indicators such as MDA, blood glucose, and body-weight loss, as demonstrated by other authors [61]. Notably, due to the scarcity of studies using *Curcuma longa* supplementation associated with physical exercise and with different types of diabetes (T1DM and/or T2DM), studies involving both humans and experimental models were considered. Biases were mainly related to the methods used both in the supplementation (doses, frequency, and duration) and in the prescription of exercise and sample population.

Some limitations must be mentioned in our work, such as the small number of articles, due in part to the strict criteria for the analysis of papers from the main databases of scientific publications (PubMed, LILACS, and Scientific Electronic Library Online). For this reason, in future studies it is necessary to find research in other scientific databases (Scopus, Web of Science, and Science direct). Other studies should be carried out to elucidate the interaction of *Curcuma longa* supplementation considering the type of diabetes, whether associated or not with different models of physical exercises, and to define the safe *Curcuma longa* concentration so that it can promote the expected antioxidant and hypoglycemic effects on the body. Food consumption should also be evaluated, which is of fundamental importance in studies involving the ingestion of substances with a potential effect on plasma parameters (e.g., blood glucose and lipid profile) when administered as a food supplement.

## 4. Conclusions

The results found in this research suggest that *Curcuma longa* supplementation has therapeutic action. The selected articles showed that turmeric had some beneficial effect on diabetes, such as better blood glucose and insulin sensitivity control, also acting as a protective factor of cells against inflammatory mediators.

Physical exercise associated with turmeric supplementation was effective in attenuating oxidative stress mediated by tissue damage markers, improving recovery after exercise and producing an antioxidant effect. These results suggest that the association of turmeric with physical exercise is promising, regarding its use for the attenuation of T1DM and T2DM effects, and studies should determine the concentration, frequency, and use of turmeric to achieve the maximum effect.

## Figures and Tables

**Table 1:** Articles selected with human and/or experimental models with different types of diabetes (Type 1 diabetes mellitus and Type 2 diabetes mellitus) submitted to turmeric supplementation and/or associated with physical exercises. Papers were analyzed and selected according to predetermined inclusion criteria.

Author and Year	Model and Blood Glucose Levels (According to Papers Analyzed)	Dose and Duration	Results
ZHAO et al. (2017) [16]	Diabetic and obese rats induced by STZ (13.88 mmol/L)	100 mg/kg body weight for 8 weeks.	↓ apoptosis in testicular cells ↓ Bax ↑ expressions of Bcl-2 ↓ MDA ↑ SOD.
YANG et al. (2018) [35]	Wistar Diabetic rats induced by STZ (≥11.6 mmol/L)	One group received 100 mg/kg of curcumin, the other received 200 mg/kg for 16 weeks.	↓ blood glucose ↓ body-weight loss
RASHID et al. (2017) [36]	Diabetic rats induced by STZ (15.5 mmol/L)	100 mg/kg of curcumin daily for 8 weeks.	↑ inflammatory cytokines, ↑ NFkB pathway translocation ↓ cytosolic NFkB expression ↑ IkBa, NFkB
SU, WANG and CHI (2017) [37]	Rats with T2DM induced by STZ (≥16.7 mmol/L)	Received medication for 8 consecutive weeks	↑ AGL and TNF-α ↓ FBG; AUCs ↓ blood glucose ↓ insulin.
PANAHI et al. (2018) [38]	Patients with T2DM (6.99 mmol/L)	Curcuminoids 500 mg/day coadministered with piperine, 5 mg/day for 3 months.	↓ insulin, HbA1c and HOMA-IR ↓ glucose and Peptide C ↓ ALT and AST
PANAHI et al. (2015) [39]	Patients with T2DM (6.1 mmol/L)	Curcuminoids 1000 mg/day + piperine 10 mg/day for 12 weeks	↓ IMC, LDL-C, CT, TG, LDL-C and non-HDL-C ↑ HDL-C
XIE et al. (2018) [40]	Sprague-Dawley rats with T1DM induced by STZ (≥11,1 mmol/L)	Treated with 1.0% curcumin (weight ratio) mixed into diet for 21 days.	↓ body-weight loss ↓ blood glucose concentration ↓ insulin concentration ↑ antioxidant genes
HARYUNA et al. (2017) [41]	Diabetic Wistar rats induced by STZ (11.1 mmol/L)	Groups 3 and 4 received curcumin therapy of 200 and 400 mg/kg for 3 days. Group 5 and 6 received 200 and 400 mg/kg for 8 days.	↑ SOD expression in cochlear fibroblasts ↓ ROS ↓ NADPH ↓ oxidase, lipoxygenase, dehydrogenase xanthine and nitric oxide synthase.
GUO et al. (2018) [42]	Diabetic Sprague-Dawley rats induced by STZ (16.7 mmol/L)	Received 300 mg/kg 16 weeks.	↓ TGF-β1, ↑ Smad7 expression ↑ AMPK; p38 and MAPK.
KANT et al. (2014) [43]	Diabetic Wistar rats induced by STZ (16.7 mmol/L)	Curcumin (0.3%) in pluronic gel once a day for 19 days.	↑ anti-inflammatory cytokine (IL-10). ↓ Ser52, GRP78, CHOP ↓ TNF-a ↑ mRNA of IL-10 ↓ IL-1b; MMP-9.
KANT et al. (2017) [44]	Diabetic Wistar rats induced by STZ (16.7 mmol/L)	0.15% curcumin topically once a day for 19 days.	↓ MDA ↑ SOD

STZ—Streptozotocin, T1DM—Type 1 diabetes mellitus, T2DM—Type 2 diabetes mellitus, IκBα—nuclear factor of kappa light polypeptide gene enhancer in B cells inhibitor alpha; NFkB—nuclear factor kappa B; BAX—associated protein X; BCL-2—B cell lymphoma protein 2; MDA—malondialdehyde; SOD—superoxide dismutase; TGF-β1—transforming growth factor beta; Smad 7—induction and downregulation; AMPK—adenosine monophosphate-activated kinase; MAPK—mitogen-activated protein kinase; HbA1c—glycated hemoglobin; HOMA-IR—homeostatic model assessment; ALT—alanine aminotransferase; AST—aspartate aminotransferase; IMC—body mass index; LDL-C—low-density lipoprotein; TC—total cholesterol; TG—triglycerides; HDL-C—high-density lipoprotein; ROS—reactive oxygen species; NADPH—the chemically reduced form of NADP; IL-10—interleukins 10; Ser52—phospho-eIF2a; GRP78—glucose-regulated protein 78; CHOP—C/EBP homologous protein; TNF-a—tumor necrosis factor; IL-1b—interleukins 1b; MMP-9—matrix metallopeptidase 9; AGL—glycosylation; FBG—fibrinogen-like; AUC—area under the curve; IL-8—interleukins 8; CK—creatine kinase; TNG—Tumor necrosis factor; sRAGE—soluble receptor for advanced glycation end-products (AGE); NEFA—nonesterified fatty acid; MDA—malondialdehyde; IL-6—interleukins 6; LV—left ventricle; ROMs—reactive oxygen metabolites; TRX-1—Thioredoxin; TBARS—Thiobarbituric acid reactive substances; GSSG—glutathione disulfide; GSH—glutathione; P38—mitogen-activated protein kinases; mRNa—Messenger RNA; Non-HDL Cholesterol; VO2 peak: maximum oxygen consumption reached before stabilization of the amount of oxygen captured; ↑—increase; ↓—decrease; =—no change.

**Table 2:** Articles selected with healthy individuals submitted to turmeric supplementation and/or associated with physical exercises. Papers were analyzed and selected according to predetermined inclusion criteria.

Author and Year	Model	Dose and Duration	Results
NICOL et al. (2015) [26]	Seventeen men	2.5 g curcumin twice a day for eccentric exercise, 2 days before and 3 days after.	↓ Ck activity ↑ IL-6 =TNF-alpha
TANABE et al. (2018) [45]	Healthy men	Group 1 ingested 180 mg/day of Curcuma 7 days before isokinetic eccentric exercise. Group 2 ingested 180 mg/day; 1 CUR 7 days after isokinetic eccentric exercise.	↑ IL-8 ↓ CK
AKAZAWA et al. (2018) [46]	Postmenopausal women	150 mg/day of curcumin along with aerobic exercise training for 8 weeks.	↑ flow-mediated dilation in postmenopausal women ↑ endothelial function
CHILELLI et al. (2016) [47]	25 healthy individuals receiving Mediterranean diet and curcumin Boswellia serrata (BSE)	12 weeks	↑ TNG ↓ sRAGE and NEFA ↓ MDA
SUGAWARA et al. (2012) [48]	Forty-five women	curcumin 150 mg/day, along with physical training with curcumin for 8 weeks.	↓ PAS ↓ ALX ↑VO_2_ peak ↓ LV afterload
TAKAHASHI et al. (2014) [49]	10 men	90 mg of curcumin 2 h before exercise and immediately after exercise for 60 min.	↑ ROMs ↑ TRX-1 =TBARS, GSSG and GSH

IL-8—interleukins 8; CK—creatine kinase; TNF—tumor necrosis factor; sRAGE—soluble receptor for AGE; NEFA—nonesterified fatty acid; MDA—malondialdehyde; IL-6—interleukins 6; LV—left ventricle; ROMs—reactive oxygen metabolites; TRX-1—thioredoxin; TBARS—thiobarbituric acid reactive substances; GSSG—glutathione disulfide; GSH—glutathione; VO2 peak: maximum oxygen consumption reached before stabilization of the amount of oxygen captured; ↑—increase; ↓—decrease; =—no change.
